# Development of an educational package for the universal human papillomavirus (HPV) vaccination programme: a co-production study with young people and key informants

**DOI:** 10.1186/s40900-022-00349-7

**Published:** 2022-04-25

**Authors:** Harriet Fisher, Tracey Chantler, Adam Finn, Joanna Kesten, Matthew Hickman, Louise Letley, Sandra Mounier-Jack, Clare Thomas, Katie Worthington, Julie Yates, Suzanne Audrey

**Affiliations:** 1 NIHR Health Protection Research Unit in Behavioural Science and Evaluation, University of Bristol, Bristol, UK; 2 NIHR Health Protection Research Unit in Vaccines and Immunisation, London School of Hygiene and Tropical Medicine, London, UK; 3 Faculty of Public Health and Policy, London School of Hygiene and Tropical Medicine, London, UK; 4 School of Cellular and Molecular Medicine, University of Bristol, Bristol, UK; 5 The National Institute for Health and Care Research Applied Research Collaboration West (NIHR ARC West), University Hospitals Bristol and Weston NHS Foundation Trust, Bristol, UK; 6 Population Health Sciences, Bristol Medical School, University of Bristol, Bristol, UK; 7 Immunisation and Preventable Diseases Division, UK Health Security Agency, London, UK; 8 Westminster House Youth Club, London, UK; 9 Screening and Immunisation, NHS England and Improvement South West, Bristol, UK

**Keywords:** Patient and public involvement, Co-production, Young people, Person-based approach, HPV vaccine, Intervention development

## Abstract

**Background:**

The English schools-based human papillomavirus (HPV) vaccination programme is routinely offered to all young people aged 12–13 years, to prevent cancers affecting the cervix, vulva, vagina, penis, anus and mouth. Lower uptake among some population groups has been identified, in part, because of unmet information needs among young people. To address these unmet needs we report intervention planning and development processes to co-produce an educational package about the HPV vaccine.

**Methods:**

We used co-production research methodologies and the ‘person-based approach’ involving the following iterative stages: (i) collating and analysing primary and secondary evidence, including HPV vaccine communication materials, interviews and workshops; (ii) developing guiding principles; (iii) undertaking a behavioural analysis informed by the Behaviour Change Wheel and the Behaviour Change Technique taxonomy; (iv) development of a preliminary logic model; (v) co-production of resources, and; (vi) refinement of resources informed by feedback from young people and key informants.

**Results:**

We co-produced EDUCATE, a theory-based educational package, that is designed to be delivered to young people prior to being offered the HPV vaccine to support uptake. Young people and key informants identified the following key issues to include as content: (i) HPV-related information; (ii) how vaccines work; (iii) safety and side-effects of the HPV vaccine; (iii) eligibility for the HPV vaccination programme, and; (iv) preparation of young people to receive the HPV vaccine. A manual for professionals (e.g. immunisation nurses, school staff) delivering the intervention and a PowerPoint presentation, interspersed with five short films, were co-produced with young people and key informants. Following feedback, the content of the EDUCATE package was refined to increase acceptability, engagement, and persuasiveness to the target users.

**Conclusion:**

Engagement with young people and key informants was integral to the development of our rigorously developed, theory-based intervention to address young people’s information needs about the HPV vaccination programme. The acceptability and persuasiveness of the package has been maximised by working closely with young people and key informants to develop the content. An implementation study to examine how the EDUCATE package is implemented in practice and the impact on uptake of the HPV vaccination programme is underway.

**Supplementary Information:**

The online version contains supplementary material available at 10.1186/s40900-022-00349-7.

## Plain English summary

In England, young people are offered the human papillomavirus (HPV) vaccine in schools at age 12–13 years to prevent some types of cancer. Some young people have unanswered questions which may stop them getting vaccinated. We report how we developed a lesson about the HPV vaccine jointly with young people and professionals to improve how information about the HPV vaccine is given. First, we reviewed research studies and HPV vaccine information materials designed for young people. Then, we carried out interviews and workshops to ask about the best way to give a lesson and the most important information to include. We worked with creative designers to produce films and animations. Finally, we asked young people and professionals for feedback on the lesson materials. The lesson includes a PowerPoint presentation with five films and guidance for people leading the session. Key topics about the HPV vaccine to be covered were identified by young people. These included information about how vaccines work and ways to improve young people’s experience of having the HPV vaccine at school. We made changes to the information in the lesson to ensure that it was relevant to young people. We are now carrying out a study to find out how well the lesson is given and whether it can help more young people get the HPV vaccine if they want it.

**Supplementary Information:**

The online version contains supplementary material available at 10.1186/s40900-022-00349-7.

## Background

The human papillomavirus (HPV) is a common infection that is spread by skin-to-skin contact, including sexual contact. Although most infections are self-limiting, infection with low-risk HPV types can lead to the development of genital warts. In rare cases, persistent infection with high-risk HPV types can lead to the development of serious health conditions affecting both women and men, including cancers of the cervix, vulva, vagina, penis, anus, and or mouth [1].

In 2008, the English HPV vaccination programme was introduced with the aim of reducing cervical cancer incidence and associated mortality [2]. The programme was first offered to young women aged 12 to 13 years through a schools-based model of delivery. To further reduce the impact of HPV-related cancers and increasing evidence of cost-effectiveness [3], from 2019/20 the programme was expanded to include young men. Through universal coverage, men who have sex with men will also receive optimal protection by immunisation ahead of sexual debut [3, 4].

The English schools-based HPV vaccination programme exceeded the 80% coverage needed to impact on future incidence of cervical cancer. However, persistent lower uptake rates among minority ethnic groups and young people living in more deprived areas have been identified [5, 6]. Our research shows complex socio-cultural factors, including information needs among young people, influence beliefs and priorities for young people to be vaccinated [7, 8].

Challenges in communicating evidence-based messages in schools-based vaccination programmes also exist. Information leaflets, together with forms requesting parental consent, are usually distributed by the school to parents or carers. Immunisation teams often have limited opportunities to interact face-to-face with young people, or to frame and target specific HPV vaccine messages to young people with additional information needs [7]. Addressing young people’s unanswered questions may help increase uptake and narrow inequalities.

The international evidence from studies undertaken in Sweden, Italy, and Australia suggests that educational interventions delivered in the school-setting can be effective at increasing young people’s understanding about the HPV vaccine [9–11], and in some cases HPV vaccination uptake [9, 10]. This suggests that an educational intervention has the potential to be effective in the English schools-based vaccination programme.

### Intervention planning and development

The Medical Research Council and the National Institute for Health Research 2021 guidance for the development and evaluation of complex interventions [12] recognises intervention development as the first of a series of interconnected steps of the development-evaluation-implementation process. Behavioural change interventions are often developed without a systematic method that draws on the evidence and theories [13]. Calls have been made for researchers to better report processes and decision-making to address under-reporting and increase clarity of intervention development [14].

This study aimed to co-produce an educational package tailored at increasing vaccine uptake in areas and populations with lower HPV vaccination coverage. At the conception stage, we drew upon our previous qualitative research findings [7] to develop an initial research plan for the co-production of an educational package that included question and answer sessions alongside a series of short videos. Co-production approaches involve sharing decision-making and including the expertise of the target users delivering and receiving an intervention [15]. By incorporating their perspectives, the acceptability, feasibility, and practicality of the intervention is addressed and maximised at the intervention development stage. Full details of the study methodology are provided in a published protocol [16].

The aim of this paper is to document the intervention planning and development processes we applied in a transparent manner. The specific objectives are to:

1. describe the process of involving target users (young people and key informants) throughout the development of the content for the educational package, and;
2. provide information about how their feedback shaped the design of the educational package.

## Methods

### Ethics

The University of Bristol’s Faculty of Health Sciences, the London School of Hygiene and Tropical Medicine's Research Ethics Committees, and the National Health Service Health Research Authority provided approvals for the study (references: 99102, 21887 & 279670).

## Research setting

Two geographically distinct areas in England with historically lower uptake of the HPV vaccination programme were selected as research sites: Bristol local authority and the London Borough of Southwark. These sites were selected as they build on existing relationships between the immunisation and research teams and where there was drive to improve delivery of the vaccination programme.

## Recruitment

Our target population groups were young men and women aged 12–15 years and key informants (e.g. immunisation nurses, youth workers and school staff). The age group 12–15 years was selected as these young people were perceived to have diverse experiences in relation to having been offered the HPV vaccine through the programme.

Prior to funding being secured, preliminary discussions around participation were held in Bristol with two youth organisations. Following research permissions being granted, information about the study was also distributed by public health officials to managers of youth organisations within Southwark, London. One organisation responded to the invitation and agreed to take part. All three organisations work with young people with sociodemographic backgrounds that have been identified with lower uptake of the HPV vaccination programme [5, 6].

Young people were initially approached by a member of staff at the participating organisations and provided with written or electronic information about the study. The research team asked the member of staff to, where possible, invite both male and female young people from different ethnic backgrounds. However, young people were not asked to provide information related to their ethnic or socioeconomic background to the study researcher. Inevitably, selection was also influenced by members of staff’s perceptions of which young people would be willing to participate and engage with the study. The recruiting process was facilitated by the good relationships that the members of staff had developed with the young people they worked with.

Key informants were identified through the research teams existing relationships with youth organisations, immunisation teams, and schools in the study areas. An additional interview was undertaken with a sex and relationships educator who was identified as the activities progressed.

In total, 61 participants took part in the research activities within the study. Some participants contributed at multiple stages.

## Intervention planning and development

We used the ‘person-based approach’ to intervention planning and development [17], which enabled us to develop an appropriate theory-, evidence-, and person-based framework to underpin the EDUCATE package. Incorporating the views of the target users (young people and professionals involved in delivery of the HPV vaccination programme) throughout the development, design, and testing processes increases the likelihood that the educational package will be acceptable, engaging, persuasive, and easy to use. In turn, this is intended to promote engagement, implementation and, ultimately, effectiveness [17].

The planning and development phases involved the following interrelated stages: (i) collating and analysing evidence; (ii) developing guiding principles; (iii) undertaking a behavioural analysis; (iv) development of a preliminary logic model; (v) co-production of the intervention, and; (vi) intervention refinement.

Further details on the methodology comprising each of these components are provided below.

### Intervention planning methodology: collating and analysing evidence

The purpose of this stage was to collate primary and secondary evidence to understand behavioural issues that lead to lower HPV vaccination uptake in the target population groups, and the challenges of addressing these.

### A scoping review of the relevant literature

As recommended by the ‘person-based approach’ [17], a scoping review was undertaken to collate evidence relating to the relevant behavioural issues. Key papers highlighting the relevant behavioural issues were identified from previous systematic reviews – supplemented by more recent work identified by the first author (HF). Additional handsearching of citations and reference lists supplemented the original documents identified.

### Patient and public involvement

We consulted with members of the Bristol Young People’s Advisory Group (YPAG) (https://generationr.org.uk/bristol/) and Bristol City Youth Council (https://www.bristol.gov.uk/youth-council-youth-mayors) about the initial design of the study. The study manager (HF) also tested the initial prototype for the intervention by delivering HPV vaccine awareness raising sessions in two schools, one of which was delivered in collaboration with an immunisation nurse.

Members of the Bristol YPAG also provided feedback on participant information sheets and recruitment materials. Feedback included that there was too much content within the information sheet, preferences for a less ‘serious’ font and inclusion of more images. Changes to the format of the documents were made according to their feedback to ensure the materials were more appealing to young people.

### Content analysis of existing HPV vaccine communication materials for young people

To inform the initial stages of the planned research, we summarised existing HPV vaccine communication materials that targeted young people and were published in the English language. The methodological approach is reported in full elsewhere [18].

### Preliminary interviews and workshops

The findings from the content analysis were used to inform topic guides to seek further feedback and clarification from young people (n = 11) and key informants (n = 6) regarding appropriate content to include within the materials for the educational package. The key areas within the topic guides were: (i) knowledge about the HPV vaccine; (ii) views of existing communication materials; (iii) delivery of educational package, and; (iv) suggestions for content of materials (e.g. participants were asked to comment on their preferences of messaging in existing HPV vaccine communication materials in relation to ‘disease that HPV can cause’, ‘side-effects’, and ‘safety’). The topic guides are provided in Additional File 1.

Subsequently, a workshop plan was developed comprising activities where 11 young people across three youth organisations were asked to: (i) review existing communication materials identified through the content analysis; (ii) comment on their understanding of key HPV vaccine messages; (iii) make suggestions of their preferred messages, design, and language style (e.g. how to communicate risk of developing cancer), and; (iv) state their preferences for how the information should be delivered (e.g. in person by a healthcare professional, through media including animation). The workshop plan is provided in Additional File 2.

Interviews and workshops were digitally recorded and conducted by two researchers (HF & TC). These were facilitated through digital platforms because of public health guidance around the coronavirus disease (COVID-19) pandemic at the time. Research activities were undertaken one-to-one, or in pairs or small groups, to suit the needs and preferences of the participants. All participants were provided with a £20 gift voucher to say ‘Thank you’ for their time.

Recordings of interviews and workshops were transcribed verbatim. Transcripts were anonymised and double checked for accuracy against the audio file. Thematic analysis [19] was undertaken assisted by the Framework approach to data management [20] within QSR NVivo software. We used both an inductive and deductive approach to analyse the content, focusing on our main research questions while identifying key issues emerging from the data. Coding of all transcripts was undertaken by one researcher (HF), while a second researcher (TC) double-coded a sub-set of the transcripts (n = 6) to check for meaning, relevance, and reliability.

Consensus meetings (HF, TC) were undertaken to review, refine, and confirm the main themes and codes relevant to the developing content for the educational package. As the process of coding progressed and data were extracted, key terms and phrases were retained while repetition and extraneous text were removed (HF).

### Intervention planning methodology: guiding principles

In the ‘person-based approach’, the guiding principles comprise a design objective and intervention features that address the user/context-specific behavioural need, issue or challenge identified in the earlier planning stages. Provisional guiding principles were iteratively developed by the research team and refined as further understanding was gained throughout the study.

### Intervention planning methodology: behavioural analysis

The behavioural analysis aimed to identify behaviours to be targeted by the EDUCATE package and their potential barriers and facilitators. These behaviours related to HPV vaccine uptake identified through the ‘collating and analysing evidence’ phase of intervention planning (See Table 1) to be targeted by the EDUCATE package and their potential barriers and facilitators. The Behaviour Change Wheel was selected as it was designed to help researchers link behaviours to inform intervention design. Identified behaviours were mapped onto constructs from the Behaviour Change Wheel [21] to clearly describe the intervention processes and components, including behavioural domains, intervention functions, and the Behaviour Change Techniques [22] to be targeted.

**Table 1:** Evidence for key behavioural determinants that the EDUCATE package is trying to address

Behavioural issue	Behavioural determinants	Evidence for behaviour	Key finding
Lower HPV vaccination uptake among young people from BAME & more deprived backgrounds	1. Low levels of understanding about the HPV vaccine among some young people	Patel H, Jeve Y, Sherman S, Moss E. Knowledge of human papillomavirus and the human papillomavirus vaccine in European adolescents: a systematic review. *Sexually transmitted infections*. 2016 Sep 1;92(6):474–9	Overall European adolescents had poor understanding of basic HPV and HPV vaccine knowledge
			Female adolescents are more likely to have heard of HPV and the HPV vaccine compared to males
			Age, higher education, and a positive vaccination status were also associated with increased awareness
		Prue G, Shapiro G, Maybin R, et al. Knowledge and acceptance of human papillomavirus (HPV) and HPV vaccination in adolescent boys worldwide: a systematic review. *J Cancer Policy* 2016;10:1–15	Globally adolescent males have poor knowledge of HPV and HPV vaccination
			Adolescent male knowledge of HPV is lower than their female peers
		Fisher H, Evans K, Ferrie J, Yates J, Roderick M & Audrey S. Young women’s autonomy and information needs in the schools-based HPV vaccination programme: A qualitative study. BMC Public Health 2020; https://doi.org/10.1186/s12889-020-09815-x	Reliance on leaflets to communicate information led to unmet information needs for young women and their families
			Almost all study participants were supportive of increasing provision of age-appropriate information for young women about the HPV vaccine. Face-to-face methods of communication were favoured
		Batista Ferrer H, Trotter CL, Hickman M, et al. Barriers and facilitators to uptake of the school-based HPV vaccination programme in an ethnically diverse group of young women. *J Public Health* 2016;38:569–77	A few of the unvaccinated young women said they had not heard about the HPV vaccine in the school setting
			Literacy and language difficulties undermine informed consent and may prevent vaccination
		Davies C, Skinner SR, Stoney T, et al. ‘Is it like one of those infectious kind of things?’ The importance of educating young people about HPV and HPV vaccination at school. *Sex Educ* 2017;17:256–75	Many young people have limited or no understanding of the vaccines they receive, including the HPV vaccine, or the diseases they are intended to prevent
	2. Reluctance to be vaccinated among young people because of fear of receiving the vaccine	Fisher H, Evans K, Ferrie J, Yates J, Roderick M & Audrey S. Young women’s autonomy and information needs in the schools-based HPV vaccination programme: A qualitative study. BMC Public Health 2020; https://doi.org/10.1186/s12889-020-09815-x	If young people are allowed to consent, some may be more likely to refuse because of fear related to being vaccinated
			Lack of priority or reluctance to receiving the HPV vaccine meant that young women could intercept the consent process, because they forgot about or misplaced the parental consent form
		Bernard D, Cooper Robbins S, McCaffery K, Scott C & Skinner S. The domino effect: adolescent girls' response to human papillomavirus vaccination. Med J Aust. 2011. 194(2011), pp.297–300	Fear of HPV vaccination was a near universal experience among adolescents in the school setting and was often associated with significant distress that had an adverse impact on the vaccination process
		Sotiriadis, A., Dagklis, T., Siamanta, V., Chatzigeorgiou, K., Agorastos, T., & LYSISTRATA Study Group. Increasing fear of adverse effects drops intention to vaccinate after the introduction of prophylactic HPV vaccine. *Archives of Gynecology and Obstetrics*. 2012: *285*(6), 1719–1724	The proportion of women rejecting vaccination for safety concerns increased significantly after the introduction of the vaccine, coinciding with isolated cases of negative publicity and highlighting the potential of misinformation by the media
		Chantler, T., Letley, L., Paterson, P., Yarwood, J., Saliba, V., & Mounier-Jack, S. (2019). Optimising informed consent in school-based adolescent vaccination programmes in England: a multiple methods analysis. *Vaccine*, *37*(36), 5218–5224	In situations where an adolescent did not want to be immunised, nurses would *‘go with the young person’* even if parents had provided consent. In cases where it was evident that the student was fearful, they would book a clinic appointment
	3. Lack of autonomy in decision-making & consent procedures by young people	Batista Ferrer H, Trotter CL, Hickman M, et al. Barriers and facilitators to uptake of the school-based HPV vaccination programme in an ethnically diverse group of young women. *J Public Health* 2016;38:569–77	The majority of vaccinated young women indicated that decisions were made by their parents, or with other adults, irrespective of their own perspective
			However, the accounts of two young women implied that they had been instrumental in ensuring that they had received the HPV vaccine after missing vaccination in the school setting
		Fisher H, Evans K, Ferrie J, Yates J, Roderick M & Audrey S. Young women’s autonomy and information needs in the schools-based HPV vaccination programme: A qualitative study. BMC Public Health 2020; https://doi.org/10.1186/s12889-020-09815-x	Where parental consent, either through paper-based consent forms or verbally, had been obtained, it was rare for young women to exercise autonomy and refuse the vaccination during the session
			Perceptions of adults as the decision-makers and targets for information, undermined opportunities for young women to be fully informed about the HPV vaccine and be involved in decisions affecting their health
		Paterson, P., Mounier-Jack, S., Saliba, V., Yarwood, J., White, J., Ramsay, M., & Chantler, T. Strengthening HPV vaccination delivery: findings from a qualitative service evaluation of the adolescent girls’ HPV vaccination programme in England. *Journal of Public Health*. 2019	Non-returned consent forms may not have been given to parents
			Students would sometimes turn up to immunisation sessions without a completed consent form
		Chantler, T., Letley, L., Paterson, P., Yarwood, J., Saliba, V., & Mounier-Jack, S. (2019). Optimising informed consent in school-based adolescent vaccination programmes in England: a multiple methods analysis. *Vaccine*, *37*(36), 5218–5224	The majority of parents (70%) said that they automatically (on receipt of the consent form) consented for their child to be immunised, with only a third of young people being involved in this decision-making

### Intervention planning methodology: logic model

In line with the Medical Research Council and National Institute for Health Research 2021 framework for the development of complex intervention [12], a logic model was developed to provide a visual representation of the proposed mechanisms of change for the EDUCATE package. This brought together the findings from the intervention planning activities and how these are anticipated to improve uptake of the HPV vaccination programme.

### Co-production of the EDUCATE resources

The next stage of the research involved co-producing the content and format of the resources being developed. Initially, researchers (HF & TC) developed preliminary written plans outlining the content and suggested structure of the educational package, which was anticipated to comprise a PowerPoint presentation integrated with short films and Question-and-Answer breaks.

The content for the films was created by young people aged 12 to 15 years (n = 16), an HPV-cancer survivor, an immunisation nurse, and the creative team at Eight Creative Agency, at Knowle West Media Centre in Bristol (www.kwmc.org.uk), who are experienced in working with young people to create health-related media materials.

This involved, three in-person filming shoots were organised across the research sites at a secondary school, a youth organisation, and an HPV-related charity. Animations were subsequently developed to include content not captured through the filming shoots. Music demos to accompany the films and animations were produced by young people enrolled on the Future Creative Leaders scheme hosted by Knowle West Media Centre. Young people were asked to provide in-person feedback on an initial story board developed by an animator and select the music to accompany each film.

A short, written training manual was produced by the study researchers (HF, TC) which contained key information, frequently asked questions. and advice as to how the package can be delivered in a way that creates a safe space for young people to ask questions (e.g. privately through a question box). Key informants (members of an immunisation team, a secondary school teacher, and professional within immunisation operations for the national programme) reviewed and commented in writing on the scripts for the videos and content of the PowerPoint presentation. This ensured the content was accurate, evidence-based, and consistent with best clinical and educational practice.

### Refinement of the EDUCATE resources

The EDUCATE package was provided to young people through a pre-recorded session delivered during tutor time in a secondary school (Bristol) and a session delivered in a youth organisation (Southwark, London). Young people (n = 17) participated in small group interviews to provide their feedback. Topic guides were developed to elicit their perceptions of the positive and negative aspects of the educational package, including how it was delivered, the design, and suggesting or creating new content. The topic guides are provided in Additional File 1.

Further feedback on the content of the educational package was obtained through a series of workshops organised with key informants (n = 15) (members of immunisation teams in both the study sites, representative from the World Health Organisation), in addition to the co-authors of this manuscript.

Responses from all participants were collated in a Table of Changes document. The researchers (HF, TC, JK) held on-line meetings to agree on modifications to the educational resources in line with the ‘person-based approach’ common guiding principles [17] and the guiding principles developed specifically for the EDUCATE package. This involved considering whether they were likely to impact on behaviour change or a precursor to behaviour change (e.g. acceptability, feasibility, persuasiveness, motivation, engagement). Prioritisation for changes were based on the MoSCoW (Must have, Should have, Could have, Would like) criteria [23]

## Findings

### Intervention planning and development activities

An overview of the results from the intervention planning and development activities are provided below.

### Intervention planning: collating and analysing evidence

#### Literature review

The literature review identified the key behavioural determinants for young people that contribute to inequalities in uptake of the English HPV vaccination programme. These include low levels of understanding about the HPV vaccine among some young people, fear of receiving the vaccine, and lack of autonomy by young people in decision-making and consent procedures (Table 1).

#### Patient and public involvement

During advisory group discussions, young people reflected on their own information needs having been offered the vaccine in the school-setting. There was consensus that videos were an appealing way to communicate health-related information. There was no clear preference for who should deliver the educational package, as both teachers and healthcare professionals were valued.

Acceptability of the initial prototype was ascertained following preliminary feedback from young people who attended sessions where it was delivered. Young people’s questions during the session related both to the HPV vaccine and preparation for vaccination. Some young people appeared reassured that the immunisation nurse delivering the information session would be present at their upcoming vaccination session, and requested that they would personally administer the vaccination. At the request of one of the schools, the information session was delivered separately by gender. This appeared to allow young men to ask different questions specifically about HPV-related cancers affecting men.

### Content analysis of existing communication materials

Overall, the communication materials identified encompassed varied formats and content, reflective of different HPV vaccination programmes and the priorities of the organisations responsible for producing the materials. For example, some communication materials presented information related to safety and side-effects in terms of how many people were anticipated to have the side-effects, whereas others only provided confirmation relating to safety or minimal side-effects with no explanatory information. Few communication materials addressed sexuality as a risk factor for HPV-related disease. We report further details of the findings elsewhere, in addition to how these were used to inform the subsequent stages of the research [18].

### Preliminary interviews and workshops: Content and delivery of the educational package

Analysis of the data focussed on key issues relevant to intervention development. These were ‘delivery’ (support, gender, addressing training needs, and role of professionals) and ‘key content to be communicated’ (risk of developing cancer, safety and side-effects, sexuality and behaviours). A summary of the findings, and illustrative quotations that were expressed concisely and typify responses relating to the themes, are presented in Table 2.

**Table 2:** Key themes from preliminary interviews and workshops

Themes & sub-themes	Key findings	Exemplar quotes
**(i) Delivery of educational package**		
Support for development	Key informants and young people were supportive of the proposed format of the educational package	‘*If it was something that was rolled out and the video gets made and there’s a resource pack, I would 99% be sure that we would use it in our session and target those the right age group… we 100% would put it on the curriculum and use it in our youth clubs*.’ [Key informant 4, male, youth worker]
	Potential to be incorporated as part of the Relationships and Sex Education (RSE) curriculum at schools or as part of the youth organisations own curriculum or programme	‘*I mean I think everybody is looking at infection and vaccines at the moment. I think everybody should be including that in their PSHE work from a science perspective, from a PSHE perspective. So, it’s a good time to produce this because vaccines are on everybody’s mind and we can kind of use it as a comparison, you know*.’ [Key informant 6, female, Sex and Relationships educator]
Gender	Perceptions of different levels of maturity by gender and the likely impact on their behaviour during the session	‘*It’s that thing that involves sex isn’t it and especially with that age group because its Year 8, so some of them know quite a lot, some of them know next to nothing and I think sometimes having the boys and girls together it turns it into that giggly messing about*.’ [Key informant 2, school medical officer]
	Most frequently young people were of the opinion that delivery should not be differentiated by gender	‘*I know probably in Year 8 I would have been fine to sit down with boys and girls but there would have been some people that wouldn’t, so it depends on the year*.’ [Young person 1, male, 15 years, workshop 1a]
	Recognition that other young people may not be comfortable asking questions in front of the opposite sex	
Addressing training needs	Immunisation nurses perceived that different levels of knowledge could hinder delivery of the educational package to young people	‘*I think a video and then maybe a one-sided A4, not teaching plan but just some points and aims that you’re trying to get out of different parts, and then maybe some questions that you can ask the young people just to get things flowing*.’ [Key informant 4, male, youth worker]
	Information provision about the HPV vaccine through a face-to-face session, or online training manual or resource pack was felt sufficient to equip professionals without specific knowledge about the HPV vaccination programme to deliver the educational package	‘*Maybe half-day training just to create awareness, give them information, more information about HPV, give them time to actually read it, have understanding about what they are passing out*.’ [Key informant 3, female, immunisation nurse]
		‘*As long as I have the information then I would be reasonably confident to go with it*.’ [Key informant 2, school medical officer]
Professional delivery of the educational package	Young people valued professionals with a medical background, who were trusted and felt to have the sufficient expertise to provide the relevant information	‘*If I was getting it [the HPV vaccine] done now and I want to speak to someone, I feel like I’d actually want to speak to people who have done it [delivered the vaccination programme] and know what it’s about*.’ [Young person 8, female, 15 years]
	Skill sets of other professionals were also recognised and felt to benefit delivery of the educational package by being able to creating a safe space to deliver the educational package in which young people felt comfortable to ask personal questions	‘*Nine times out of 10 there’s a teacher or that one person [in the school setting] that everyone’s comfortable talking about stuff with and they’re the sort of person then that will be able to shut it down and be more like come on guys we’re having a laugh now … but not shut them down and say we’re not going to talk about it*.’ [Key informant 4, youth worker, male]
	Some young people recognised the relationships they had with particular professionals would help facilitate more open discussions	‘*If they [immunisation nurse] were linked up with a youth worker and they worked well together and could present parts of it each then they might be more… they might be quite willing to ask questions to youth workers then*.’ [Key informant 1, immunisation nurse, female]
		‘*If [Sex and Relationships Educator] came into school and delivered a session about it I would feel really comfortable with her*.’ [Young person 1, female, interview]
**(ii) Communication of content within the educational package**		
Risk of developing cancer	Personal experiences of HPV-related cancer could provide a powerful message to be vaccinated	‘*If you said 8,000 people in the UK I’d think that still makes that young person go ‘well that’s not going to be me’ you know what I mean like there are 66 million people in this country you know what I mean if they’re good with numbers they’ll know that and they’ll be like that’s not going to be me*.’ [Key informant 4, youth worker, male]
	Presentation of information related to the annual incidence of cervical cancer was not felt to be an effective way to communicate risk with young people	‘*I think just seeing seven out of 10 people [acquire HPV over their life-time], that means like, three people won’t get it and that’s quite—that’s a very small number not being affected by this and it would make me think I may as well go for it*.’ [Young person 1, male, workshop 1a]
	Information related to the prevalence of HPV was felt to be a more effective message than providing data related to incidence of cancer	‘*Personally I would expect people who have actually gone through the cancer that the boys or girls could get… so I think that would be quite good because you could see what they’ve been through… then you can see… oh I should get it because I don’t want that happening to me*.’ [Young person 8, female, 15 years old]
		‘*I think if I’ve got someone in my family or I myself had the cancer and someone stood up and said, ‘let's hear this person talk about their story,’ sitting there watching someone else talk about their story, although it might make me very sad it might also make me very hopeful*.’ [Young person 1, male, workshop 1a]
Safety and side-effects	Presenting information on rare but serious side-effects could adversely affect young people’s decision-making about having the HPV vaccine	‘*I think it’s [information about serious side-effects] more of something that you’d have to say to their parents because I feel like, if you’d said that to me when I was 12 or 13 I’d probably stay very clear away from it*.’ [Young person 1, male, workshop 1a]
		‘*That might be one thing that would scare somebody and put them off more than it would want to have it done actually, oh my god I’m going to have a shock and it’s going to stop my heart*.’ [Key informant 4, youth worker, male]
Sexuality and behaviours	Young people and key informants supported that young people should be told how HPV is transmitted and who is a greater risk	‘*I work with someone at the minute who’s 13 and has come out as gay already so it’s good information for them to know that they’re at risk as well*.’ [Key informant 5, male, youth worker]
	Mixed levels of understanding and embarrassment among vaccine eligible young people and the school environment does not facilitate in-depth conversations or open discussions	‘*I think it would have to be very basically written because I think some of the Year 8 boys that I’ve come across have no idea that that is even a possibility and whether parents would take offence at us being the person to broach that with them that this is a possibility. But then there are boys on their, you know, in Year 8, you know, especially if they’re considering that they may be gay that this is something that they are well aware of and it is something they have thought about*.’ [Key informant 1, female, immunisation nurse]

### Intervention planning: guiding principles

In brief, the EDUCATE package aims to: (i) improve young people’s knowledge and understanding about the HPV vaccine; (ii) increase young people’s confidence to have a vaccine in the school setting; (iii) engage young people in the decision-making and the consent process, and; (iv) be delivered flexibly to meet the needs of the target population (Table 3).

**Table 3:** Guiding principles for the EDUCATE package —an intervention to improve HPV vaccine uptake

Design objectives that address each key issue	Key intervention features relevant to each design objective
Improve young people’s knowledge and understanding about the HPV vaccine	Provide information in a format appealing to young people about:
	(i) how vaccines work
	(ii) HPV & HPV-related illnesses
	(iii) HPV vaccine
	(iv) HPV vaccination programme
	(v) preparation
	(vi) getting the vaccine
	Persuasive content of educational package highlighting benefits to increase motivation to be vaccinated (e.g. case-study of HPV-related cancer survivor)
Increase young people’s confidence to have a vaccine in the school setting	Educate young people about the safety profile of the HPV vaccine
	Normalise vaccination process (e.g. examples of young people’s talking about their experiences, filming of school-based vaccination session)
	Acknowledge some young people have anxieties about receiving the vaccine
	Suggest ‘coping strategies’ to young people to improve experience of having the vaccine
	Provide a safe space to address young people’s concerns about receiving the HPV vaccine (e.g. side-effects, anticipated pain)
Engage young people in decision-making and the consent process	Provide young people with parental consent forms and clear instructions about how to get the HPV vaccine at school during educational session when motivation is highest
	Educate / signpost young people about availability of vaccine in different settings
Be delivered flexibly to meet needs of target population	Deliver separately by gender if advised by school
	Q&As from young people during the session can be interspersed during session, or delivered at the end. Use of ‘question box’ can provide young people opportunity to ask confidential questions without embarrassment
	Choice of person to deliver the session (e.g. immunisation nurse, youth worker, school staff)
	Tailoring content of PowerPoint to be applicable to local context (e.g. self-consent procedures, missed doses)
	Use of audio-visual communication materials in formats and styles appealing to young people

### Intervention planning: behavioural analysis

The proposed EDUCATE package employs four intervention functions (enablement, education, persuasion, and environmental restructuring) which are enacted by six behavioural change techniques (‘instruction on how to perform a behaviour’, ‘information about health consequences’, ‘anticipated regrets’, ‘generalisation of target behaviour’, ‘pros and cons’, and ‘restructuring the social environment’) (Table 4).

**Table 4:** Behavioural analysis of EDUCATE intervention using the Behaviour Change Wheel and the Behaviour Change Technique taxonomy

Target behaviour	Barriers to target behaviour	Intervention strategy	Intervention function [1]	Behavioural change technique [2]
Increase uptake of the HPV vaccine	Limited knowledge and understanding about the HPV vaccine among young people	Persuasive content of educational package highlighting benefits to increase motivation to be vaccinated (e.g. case study of HPV-related cancer survivor)	Enablement *(increasing means/reducing barriers to increase capability or opportunity)*	4.1 Instruction on how to perform a behaviour
	Lack of motivation and interest to be vaccinated	Clear, age-appropriate content within communication materials to ensure understanding	Education *(increasing knowledge or understanding)*	5.1 Information about health consequences
	Fear of vaccination	Use of audio-visual communication materials in formats and styles appealing to young people	Persuasion *(using communication to induce positive or negative feelings or stimulate action)*	5.5 Anticipated regret
	Lack of young people’s engagement with decision-making and consent process	Content focussed on increasing knowledge and motivation to be vaccinated through:	Environmental restructuring *(changing the physical or social contact)*	8.6 Generalisation of target behaviour
		Explanation of how vaccines work, HPV, related illnesses, HPV vaccine & HPV vaccination programme		
		Examples of other young people, and their experiences of, receiving the HPV vaccine		9.2 Pros and cons
		Suggest ‘coping strategies’ to young people to improve experience of having the vaccine		12.2 Restructuring the social environment
		Provide a safe space to address young people’s concerns about receiving the HPV vaccine (e.g. side-effects, anticipated pain)		
		Provision of parental consent forms and clear instructions during educational session when motivation is highest		
		Educate / sign post young people about availability of vaccine in other settings		

### Intervention planning: logic model

**Fig. 1:**
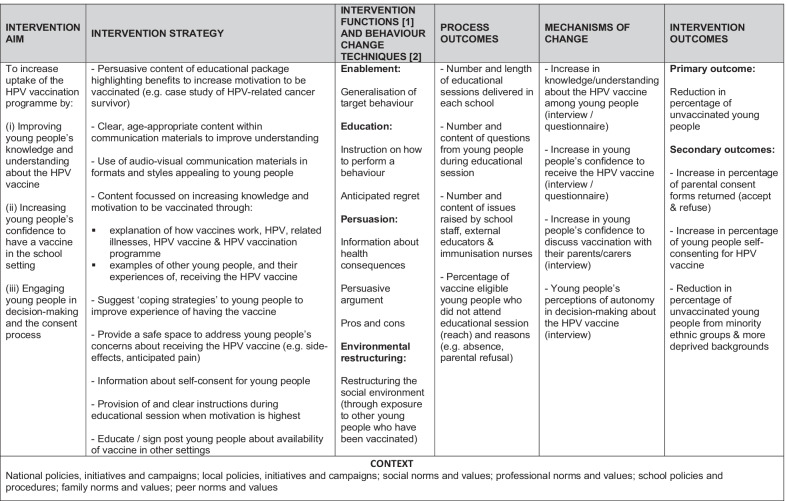
Logic model for EDUCATE

The logic model for the EDUCATE is provided in Fig. 1.

### Co-production of the EDUCATE resources

Building on our findings from the interview and workshop data with young people and key informants, and the wider literature, the following key themes for content were selected: (i) HPV-related information; (ii) how vaccines work; (iii) safety and side-effects of vaccination; (iv) eligibility for the HPV vaccination programme, and; (v) preparation of young people to receive the HPV vaccine [18]. Signposting young people to other information sources, such as websites or professionals involved in the delivery of the programme, was also valued.

Film shoots were organised around the following scenarios confirmed by young people: (i) interview with young people post-vaccination around their experience of having the vaccine at school; (ii) unvaccinated young people interviewing vaccinated (older) young people; (iii) young people interviewing healthcare professionals to find out key information; (iv) a vaccination session taking place, and; (v) a case study with a person who has experienced HPV-related cancer. Building on feedback from young people expressing the importance of feeling represented in the materials, participants were ethnically diverse and filming took part in different settings (e.g. schools, youth clubs).

An initial script and story board for an animation was developed to capture content not captured through the filming shoots (‘HPV-related information’ and ‘how vaccines work’). Feedback from young people resulted in changes related to communicating messages around: the sexual transmissibility of HPV; ensuring imagery of young people were ethnically diverse, and; avoiding assumptions around HPV infection risk only affecting heterosexual partnerships.

There is a wide range of maturity, knowledge and experience in relation to young people’s sexual development aged 12–13 years. For example, some of the target population may already be aware of their own sexuality or sexual preferences, or be sexually active. Other young people may become interested in relationships and sex at an older age. There was consensus from young people and key informants that it was important to be honest and not withhold information related to sexual behaviours from young people as part of this educational resource. Therefore, content related to key issues regarding sexual behaviours and sexuality in relation to the HPV vaccine have been included (e.g. higher risk of developing HPV-related cancers among men who have sex with men). However, the content was deliberately kept brief or included only withing the training manual so young people would not be overloaded with information if they are not emotionally ready for it.

To ensure applicability to wider population groups with different cultural values or belief systems, the EDUCATE resources were developed as a universal package that primarily focuses on ‘health prevention’ rather than focussing on ‘sex education’. The content related to the sexual transmission of HPV is minimal. Further, the resources were not translated into additional languages as they have been designed to be delivered in settings (e.g. English schools) where sessions are delivered in English.

### Refinement of the EDUCATE resources

The resources for the EDUCATE package are PowerPoint slides, interspersed with five short films. A guidance document with additional information for the delivery of the session was also produced. We intend to make the final product available in the public domain shortly.

The creative team selected a colour palate and font that was intended to be appealing to young people. Overall, young people and key informants were positive about the materials and felt they would be beneficial in helping young people find out about the HPV vaccine.

The changes enacted upon were mostly minor alterations to the content of the PowerPoint slides, such as changing words to improve clarity of meaning. In some cases, further content was added to the guidance document to balance providing additional information within the PowerPoint with overloading information at the risk of disengaging young people (e.g. availability of HPV vaccine for men-who-have-sex-with-men in sexual health and HIV clinics). Additional notes to assist delivery of the package were added to the PowerPoint slides. Design changes included the redesign of existing diagrams to match the overall style and ensuring gender balance of figures on the PowerPoint slides. The length of one of the films was also reduced to ensure the information was succinct.

## Discussion

This manuscript reports the systematic theory, evidence, and person-based approach [17] we used to co-produce the EDUCATE package. The resources comprise a training manual and PowerPoint presentation, interspersed with five short films. The EDUCATE package is intended to be used alongside, and complement, materials developed for the national HPV vaccination programme. Finally, it has been specifically designed to address information needs of young people less likely to be vaccinated.

We collaborated with young people, and key informants (including school staff, immunisation teams, public health staff, and multi-disciplinary academics) at all stages of the research to iteratively optimise the content and design of the intervention to increase acceptability to the target user groups. The Behaviour Change Wheel [21] was used to underpin the EDUCATE package theoretically and to identify the target Behaviour Change Techniques [22].

Within healthcare research, there has been an increase in recent years of the use of co-production methodologies, that involve sharing decision-making and including the expertise of the target users delivering and receiving an intervention [15]. Examples of co-production studies involving young people include informing the redesign of an educational psychology service [24] and the design of a social media suicide prevention campaign [25]. However, we are unaware of any interventions to address adolescent vaccination uptake and inequalities that have been developed using the principles of co-production. The EDUCATE package therefore addresses this evidence gap.

As part of this study, we enjoyed working closely with young people from disadvantaged backgrounds who are often under-represented in research. Ensuring their voices were heard throughout this study was further challenged by the on-going COVID-19 pandemic, which included at times closures of youth organisations and the necessary reliance on digital platforms to conduct research among a population group with limited access. However, we were able to overcome these barriers through the perseverance and willingness of professionals to facilitate the research activities and young people to be involved.

Despite the increased popularity of using co-production methodologies to develop interventions, the effectiveness on long-term changes in population health behaviours are currently uncertain [26]. To address this research gap, we plan a future implementation study to further test the mechanisms of delivery (e.g. engage young people in the decision-making and the consent process) and gather evidence in relation to changes to uptake of the HPV vaccination programme.

## Limitations

We were unable to conduct further planned piloting of the EDUCATE package, because of COVID-19 restrictions requiring young people at a second youth organisation to self-isolate. There were also time constraints caused by further engagement activities taking longer to organise because of extra demands on school staff and immunisation teams caused by responding to changing demands of the pandemic response. Further, the views expressed by contributors may not be generalisable to other groups of young people less likely to receive the HPV vaccine. For example, young people with Special Educational Needs or Disability were not represented. Information related to the ethnic or socioeconomic background of young people who participated in the study was not collected. The role of parents, and their information needs, were not considered as part of this study.

## Conclusion

This study has begun to address the need for a rigorously developed, theory-based intervention to meet young people’s information needs about the HPV vaccination programme. By working closely with young people and key informants, the content of the communication materials was developed to meet the needs of target users. Future studies to examine how the EDUCATE package is implemented in practice and the impact on uptake of the HPV vaccination programme are underway.

## Supplementary Information

**Additional file 1: **Topic guides.

**Additional file 2: **Workshop plan.

## Data Availability

It is the authors’ intention to share their underpinning research data in order to maximise reuse and evidence their findings. When participants consent or assent to take part in the study, their permission to deposit anonymised transcripts at the University of Bristol Research Data Repository (data.bris.ac.uk/data) was sought. A metadata record will be published openly by the repository and this record will clearly state how data can be accessed by bona fide researchers. Requests for access will be directed to the Research Data team at Bristol, who will assess the motives of potential data re-users before granting access to the data. No authentic request for access will be refused and re-users will not be charged for any part of this process.

## Abbreviations

COVID-19: Coronavirus disease
HPV: Human papillomavirus
MoSCoW: Must have, should have, could have, would like
YPAG: Young Person’s Advisory Group
