# Supplementary material for: Development of an educational package for the universal human papillomavirus (HPV) vaccination programme: a co-production study with young people and key informants
Source: Res Involv Engagem. 2022 Apr 25;8:16. doi: 10.1186/s40900-022-00349-7 (PMC9035505; doi:10.1186/s40900-022-00349-7)
Supplement: Supplementary file 1 — Additional file 1: Topic guides. [file 40900_2022_349_MOESM1_ESM.docx]

**Stage One: Young Person Topic Guide**

[Note: *Ensure that the participant has read the information sheet and signed the assent form before the interview starts*.]

We are interested in finding out the best ways of helping young people find out about the HPV vaccine.

Do you use social media at all?

If yes, can you tell me a little bit more about how you use it?

What platforms do you use?

What do you like about it?

Is there anything you dislike about it?

How do you think it could be improved?

Do you watch videos on social media at all?

If yes, can you tell me a little bit more about how you use it?

Can you tell me about some videos that you have seen recently?

What do you like about it?

Is there anything you disliked about it?

Can you think of any ways it could be improved?

Before this study, had you heard about the HPV vaccine?

If yes, can you remember how you found out about it?

Can you remember what information were you given?

Was there any other information you wanted?

Was this a good way to find out about the vaccine?

Can you think of any better ways of finding out about the HPV vaccine?

If you wanted to find out about the HPV vaccine now, how do you think you would do this?

Do you think a leaflet is a good way of giving information?

Can you remember if you watched any videos about the HPV vaccine?

If yes, can you tell me a little bit more about that?

Where did you watch the video?

What did you like about it?

Was there anything you didn’t like about it?

How could we improve it?

Show example of universal HPV vaccination programme leaflet and allow participant to read the information.

1. Overall, what did you think about the leaflet?
2. Was the leaflet difficult or easy to understand?
3. If you were offered the HPV vaccine, what do you think the most important messages would be for you?
4. Do you think anything could be done differently?
5. Is there any other information you think is important to be included?
6. The HPV vaccine is available free of charge in England until young people are aged 25 years old. Is this important information to include?
7. The HPV vaccine leaflet for England shows young people in school uniforms. Do you think this is a good thing or not?

Do you think a lesson in school is a good way of helping young people find out about the HPV vaccine, or not?

Why do you think about that?

If yes, who would you like to deliver a lesson?

Teachers

Other members of staff at school

Youth workers

Immunisation nurses

Some of the information given in a lesson like this might talk about how HPV affects young men and women’s different body parts.

How comfortable do you think you would feel to ask questions if the session was delivered to:

- your entire year group?

- your tutor group / class?

- mixed gender groups

- boys and girls separately?

Do you think videos are good ways of helping young people find out about the HPV vaccine, or not?

Why do you think about that?

If there were videos about the vaccine, what sort of things do you think are the most important things are to include?

Vaccines

Diseases that HPV can cause

- Should we present this information separately by gender or not?
- Is it helpful to know how many people develop the condition each year? E.g. 7,000 women develop cervical cancer each year in England; About 2,000 men a year in the UK develop a cancer caused by HPV and some of these cancers are becoming more common

Side-effects

- What information would you need to know?
- Very common side-effects that affect more than 1 in 10 people
  - These include redness at site, swelling, pain, headaches
- Less common side-effects that affect less than 1 in 1000 people
  - These include bruising, temperature, nausea
- Rare side-effects that affect less than 1 in 100,000 people
  - This could be having an allergic reaction to the vaccine

Safety

- What information would you need to know?
- How many people have had the vaccine?
  - 80 million people world wide
  - 10 million doses in England
- Research evidence that the vaccine is safe?
- How data about safety is regularly monitored in England and worldwide
- How immunisation nurses keep you safe?

Monitor you after having the vaccine

- - Training in case you have a reaction

Consent procedures

Young people talking about their experiences

Seeing someone receive the vaccine

- School setting? Doctors surgery What to expect on the day

What you need to do

What format do you think they should be?

e.g. animations, vloggers, interviews with immunisation nurses or doctors

Who should take part in the video?

Prompt (Immunisation nurse. Doctor? Teacher. Youth worker. Parent. Young people)

Would you like to see-

Someone receiving the vaccine

Immunisation nurse giving information

Young people talking about their experiences

Whether it hurts or not?

What to expect on the day

Where the vaccine is given; Questions the immunisation nurses will ask you; What the sensation of receiving the vaccine? What happens when after you have had the vaccine?

What you need to do

Consent procedure

**Finally, is there anything else you would like to tell me or ask me about?**

**Many thanks for taking part in this interview.**

[NOTE: *ensure that the participant is given the £20 gift voucher*]

**Stage One: Professional Topic Guide**

[Note: *Ensure that the participant has read the information sheet and signed the consent form before the interview starts*.]

We are interested in finding out the best ways of helping young people find out about the HPV vaccine.

Do you use social media at all?

If yes, can you tell me a little bit more about how you use it?

What platforms do you use?

What do you like about it?

Is there anything you dislike about it?

How do you think it could be improved?

Do you watch videos on social media at all?

If yes, can you tell me a little bit more about how you use it?

Can you tell me about some videos that you have seen recently?

What do you like about it?

Is there anything you disliked about it?

Can you think of any ways it could be improved?

Can you tell me how you think young people find out about the HPV vaccine?

Can you think of any better ways for young people to find out about the HPV vaccine?

Do you think a leaflet is a good way of giving information?

Have you ever watched any videos about the HPV vaccine?

If yes, can you tell me a little bit more about that?

Where did you watch the video?

What did you like about it?

Was there anything you didn’t like about it?

How could we improve it?

Show example of universal HPV vaccination programme leaflet and allow participant to read the information.

1. Overall, what did you think about the leaflet?
2. Was the leaflet difficult or easy to understand?
3. If you were offered the HPV vaccine, what do you think the most important messages would be for you?
4. Do you think anything could be done differently?
5. Is there any other information you think is important to be included?
6. There are some groups of young people at greater risk of HPV related disease (e.g. men who have sex with men). Do you think this information should be included?
7. The HPV vaccine is available free of charge in England until young people are aged 25 years old. Is this important information to include?
8. There is no age limit for men who have sex with me to have the HPV vaccine. Should this be included?
9. The HPV vaccine leaflet for England shows young people in school uniforms. Do you think this is a good thing or not?

Do you think a lesson in school is a good way of helping young people find out about the HPV vaccine?

Why do you think that?

If yes, who do you think should deliver a lesson?

Teachers

Other members of staff at school

Youth workers

Immunisation nurses

Would you feel confident to deliver a lesson about the HPV vaccine?

Why is that?

Would you require any training to deliver a lesson about the HPV vaccine?

If yes, how should the training be delivered?

What are the most important aspects that should be covered?

Some of the information given in a lesson like this might talk about how HPV affects young men and women’s different body parts.

How comfortable do you think young people would feel to ask questions if the session was delivered to:

- an entire year group?

- a tutor group / class?

- mixed gender groups

- boys and girls separately?

Can you think of any other ways we can help young people find out about the vaccine?

Do you think videos could help young people find out about the HPV vaccine, or not?

Why do you think that?

If there were videos about the vaccine, what sort of things do you think are the most important things are to include for young people?

Vaccines

Diseases that HPV can cause

Side-effects

- Should we present this information separately by gender or not?
- Is it helpful to know how many people develop the condition each year? E.g. 7,000 women develop cervical cancer each year in England; About 2,000 men a year in the UK develop a cancer caused by HPV and some of these cancers are becoming more common
- What information would you need to know?
- Very common side-effects that affect more than 1 in 10 people
  - These include redness at site, swelling, pain, headaches
- Less common side-effects that affect less than 1 in 1000 people
  - These include bruising, temperature, nausea
- Rare side-effects that affect less than 1 in 100,000 people
  - This could be having an allergic reaction to the vaccine

Safety

- What information would you need to know?
- How many people have had the vaccine?
  - 80 million people world wide
  - 10 million doses in England
- Research evidence that the vaccine is safe?
- How data about safety is regularly monitored in England and worldwide
- How immunisation nurses keep you safe?

Monitor you after having the vaccine

- - Training in case you have a reaction

Consent procedures

Young people talking about their experiences

Seeing someone receive the vaccine

School setting? Doctors’ surgery

What to expect on the day

What you need to do

What format do you think they should be so they are relevant to young people?

e.g. animations, vloggers, interviews with immunisation nurses or doctors

Who should take part in the video?

Prompt. Doctor? Immunisation nurse. Teacher. Youth worker. Parent. Young people

Do you think young people would like to see:

Someone receiving the vaccine

Immunisation nurse giving information

Young people talking about their experiences

Whether it hurts or not

What to expect on the day

Where the vaccine is given; Questions the immunisation nurses will ask you; What the sensation of receiving the vaccine? What happens when after you have had the vaccine?

What you need to do

Consent procedure

**Finally, is there anything else you would like to tell me or ask me about?**

**Many thanks for taking part in this interview.**

[NOTE: *ensure that the participant is given the £20 gift voucher*]

**Stage Three: Young People’s Topic Guide**

**Explain purpose of project and session.**

[Note: *Ensure that the participants have read the information sheet and signed the consent form before the discussion starts*.]

I’d like to hear about your views and experiences about the lesson about the HPV vaccine. This is very informal; you can talk about anything you think is important for us to know. Remember, your answers to our questions will not be considered “right” or “wrong”, because I want to know about what you think.

- Explain the conversation is being recorded.
- Answer any questions they may have.
- Ensure either written or verbal parental consent and adolescent assent is obtained from the participant.

**The EDUCATE lesson**

The EDUCATE lesson was designed to be delivered to young people before they are offered the HPV vaccine. It aims to help young people make more informed decisions by equipping young people with knowledge about the HPV vaccination programme and providing reassurance about receiving the vaccine at school.

We developed a PowerPoint presentation with five short films that you have seen. We designed the lesson specifically to address the information needs of young people from populations where coverage is low.

**HPV vaccine**

- Before you were attended the lesson, had you ever heard of HPV?
- Can you tell me about what you know about HPV now?
- Can you remember what information were you given?
- What do you think are the positive things about the HPV vaccine are?
- What do you think the negative things about the HPV vaccine are?

**Overview**

- Overall, what do you think about the lesson that you saw at [name of organisation]?
- What are your reasons for this?
- Which part of the lesson did you like most? Why?
- Were there any parts of the lesson that didn’t like? What could we change?
- Is there any other information you would have liked?

**Delivery of the lesson**

Can you tell me about how the lesson delivered?

- Pre-recorded session vs. face-to-face delivery
- During lesson / assembly / tutor time
- Single vs. multiple sessions
- Preference for the session to be delivered separately by gender

Can you remember how long the session/s lasted for?

Can you tell me about who delivered the lesson?

- Did you know them already?
- How knowledgeable do you feel that person was about the HPV vaccine?
- Is there anyone else you would have preferred to deliver the session? (e.g. nurse, school staff member)

Did anyone ask any questions during the lesson?

- How did students ask questions (e.g. question box, hands up)
- Can you remember what the questions were?
- Was the teacher/nurse able to answer the questions?
- Did you have any questions you wanted to ask during the lesson?
- How confident did you feel to ask the questions? Why was that?
- Could this be improved?

**Decision-making and consent** [QUESTIONS MAY NOT ALL BE APPLICABLE]

How did you give consent to have the vaccine or not?

Did the school provide you with consent forms at all?

If yes, when did they give you the consent forms?

Who decided whether you should have/not have the vaccine?

Who helped with the decision? Prompt: Mother? Father? Other family members? Friends? Teacher? School nurse?

- What did they say?
- Did they agree whether you should have the vaccine or not?
- How confident did you feel to talk to your them about the HPV vaccine?
- Was there anything you did not feel confident talking to them about?

After the lesson, do you think you had enough information to decide whether to have the vaccine or not after the lesson?

Before the lesson, did you know young people are able to give their own permission to have the HPV vaccine?

Do you think you had enough information to do that?

**Receiving the HPV vaccine**

Did you receive the HPV vaccine? If yes, how did you feel?

Did you have any concerns or worries? If yes, what were these?

How prepared did you feel to have the HPV vaccine after this lesson?

**Feedback on communication materials**

Ask the participant what they thought on different following components of the package.

- *PPT content: All about HPV*
- *Film 1. HPV and the immune system*
- *PPT content: HPV-related illnesses*
- *Film 2. Case-study of HPV-related throat cancer survivor*
- *PPT content: HPV vaccine*
- *PPT content: HPV vaccination programme*
- *Film 3. Questions and answers with an immunisation nurse*
- *PPT content: Preparation*
- *Film 4. Strategies to help on the day of the vaccination session*
- *PPT content: Getting the vaccine*

*Question prompts: Films*

- Overall, what do you think about the film about X?
- What do you like about the film?
- Is there anything you do not like about the film?
- What message do you think the film was trying to communicate?
- How relevant do you think the messages of the film are to young people?
- Can you think of any other messages or content that could be included?
- Is there any messaging or content that you think could be left out?

What do you think of the style?

Tone - humour / serious?

Representation of people? (gender, ethnicity, sexuality, settings)

*Question prompts: PPT*

- What message do you get from the content about X? Anything unclear?
- Feedback on content of PPT?
- Is the content of the PPT difficult or easy to understand?
- Is there any additional content on this topic important to include?
- Any messaging or content that you think could be left out?

What do you think about the font or typology?

Bigger? Smaller?

Does the colour stand out enough?

Is it easy to read?

**Finally, is there anything else you would like to tell me or ask me about?**

**Many thanks for taking part in this interview.**

[NOTE: *ensure that the participant is given the £20 gift voucher*]

**Stage Three: Professional Topic Guide**

**Explain purpose of project and session.**

- We are part of a team working on the EDUCATE study which aims to co-produce an educational package aims to address young people’s unmet information needs and improve uptake of the HPV vaccination programme.
  - For the study we have developed communication materials which can be delivered as part of a session about the HPV vaccine, either in the school setting or youth organisations
  - The communication materials have been developed in collaboration with the creative team at Knowle West Media Centre, young people and key stakeholders
  - We are now seeking feedback from young people and key stakeholders to ensure that the package is fit for purpose
  - We anticipate that the session will last up to two hours (workshop) or 45 minutes (interview)

**Aim**

- Today, we would like to ask for your feedback on the communication materials we have developed as part of the EDUCATE package
- Explain the conversation is being recorded so the researcher present can make notes after.
- Answer any questions they may have.
- Ensure either written or verbal consent is obtained from the participant.

**The EDUCATE package**

The educational package has been designed to be delivered to young people before they are offered the HPV vaccine. It aims to help young people make more informed decisions by equipping young people with knowledge about the HPV vaccination programme and providing reassurance about receiving the vaccine at school.

We developed an introduction manual for staff delivering the programme and a PowerPoint presentation, interspersed with five short films. There is also time allocated for Questions and Answers. The communication materials for the educational package have been designed to specifically address the information needs of young people from populations where coverage is low.

We think the educational package will be most useful if it is delivered to Year 8 students before they are offered the HPV vaccine. However, other young people may also benefit from having the opportunity to find out more about the HPV vaccine. Different professionals, such as school staff, youth workers, immunisation nurses, or school nurses, could deliver the educational package.

- Overall, what do you think about using the educational package in this way?
- What are your reasons for this?

**PART 1**

**N.B. These questions about delivery of the package are only applicable if the professional was involved in organising or delivering the EDUCATE package. Skip to Part 2 if not applicable.**

**Background questions**

What is your current job title and role in the [name of organisation]?

How long have you worked at this organisation?

**HPV vaccine**

What do you know about the HPV vaccine?

What is your opinion about delivering educational session about the HPV vaccine in schools or other settings?

Prompt: Who is responsible for this? Positive and negative aspects of delivering the package in this setting.

Normally, how are Year 8 students informed out about the HPV vaccination programme?

How are their parents/carers informed about the HPV vaccination programme?

**Delivery of the session**

How was the session delivered?

- **Prompts:** Assembly, tutor time, separately by gender, class size, multiple sessions

Overall, how easy was it to organise deliver of the session?

Approximately how long did the session/s last for?

How confident did you feel to deliver the session?

- Were the materials provided by the research team sufficient?

**Prompts:** PPT document, Introduction document, examples of FAQs

- Would additional training have been helpful?

How do you think young people responded to the way the session was delivered?

- Pre-recorded session vs. face-to-face delivery
- Professional delivering the session (e.g. school staff member, immunisation nurse)
- Which part do you think the young people responded best to?
- Which part do you think the young people responded least well to?
- Were there any groups of young people you felt were unable to engage with the session fully (e.g. cultural barriers)
- Do you think this could be improved in anyway?

How were young people’s questions managed during the session? (e.g. question box, hands up)

- Did young people have their questions answered during the session?
- Can you remember what they were?
- Did you feel equipped to address these?
- Do you think issues around confidentiality were addressed sufficiently?
- Could this be improved?

Were young people provided with parental consent forms during or directly after the session?

- Is this different to how they would usually be informed about consent procedures?
- How engaged did the young people appear with the process?

**Following delivery of the session**:

- Did any parents get in touch with you about the educational session?
- Did any young people approach you about the HPV vaccine following the session? What questions did they ask you?
- How confident do you think young people would feel to discuss having the vaccine with their parents?
- Do you think young people were more or less likely to return their consent forms to the school?
- Do you think young people are more or less likely to be vaccinated following the lesson?
- How prepared do you think young people were before having the vaccine at school?

Thinking about delivery of the educational package:

- Overall, what do you think has worked well?
- Overall, what do you think hasn’t work so well?

Do you have any suggestions for improvements to how the educational package is delivered? If yes, what?

**PART 2**

**If the participant was not involved in the delivery of the EDUCATE package, explain that we are going to run through communication materials as they would be used in a session delivered to young people.**

**If the participant was involved in the delivery of the EDUCATE package, ask them to provide feedback on the different components of the package. Show the participant the materials again if necessary.**

**Ask the participant to provide a commentary on the following components of the package.**

- *PPT content: All about HPV*
- *Film 1. HPV and the immune system*
- *PPT content: HPV-related illnesses*
- *Film 2. Case-study of HPV-related throat cancer survivor*
- *PPT content: HPV vaccine*
- *PPT content: HPV vaccination programme*
- *Film 3. Questions and answers with an immunisation nurse*
- *PPT content: Preparation*
- *Film 4. Strategies to help on the day of the vaccination session*
- *PPT content: Getting the vaccine*

*Question prompts: Films*

- Overall, what do you think about the film about X?
- What do you like about the film?
- Is there anything you do not like about the film?
- What message do you think the film was trying to communicate?
- How relevant do you think the messages of the film are to young people?
- Can you think of any other messages or content that could be included?
- Is there any messaging or content that you think could be left out?

What do you think of the style?

Tone - humour / serious?

Representation of people? (gender, ethnicity, sexuality, settings)

*Question prompts: PPT*

- What message do you get from the content about X? Anything unclear?
- Feedback on content of PPT?
- Is the content of the PPT difficult or easy to understand?

Prompt: Staff. Young people.

- Any content that you don’t agree with?
- Was the content appropriate for all groups of young people (e.g. cultural preferences)
- Is there any additional content on this topic important to include?
- Any messaging or content that you think could be left out?

What do you think about the font or typology?

Bigger? Smaller?

Does the colour stand out enough?

Is it easy to read?

**Overall**

What did you think of the educational package?

Are there any improvements we could make?

Are there any additional content or messages that you think are important to include?

What did you think about the order the content was presented?

Are there any other routes to sign post young people that should be included?

Are you happy for your organisation’s logo to be included? [IF APPLICABLE]

**Finally, is there anything else you would like to tell me or ask me about?**

**Many thanks for taking part in this interview.**

[NOTE: *ensure that the participant is given the £20 gift voucher*]
