# Supplementary material for: Development of an educational package for the universal human papillomavirus (HPV) vaccination programme: a co-production study with young people and key informants
Source: Res Involv Engagem. 2022 Apr 25;8:16. doi: 10.1186/s40900-022-00349-7 (PMC9035505; doi:10.1186/s40900-022-00349-7)
Supplement: Supplementary file 2 — Additional file 2: Workshop plan. [file 40900_2022_349_MOESM2_ESM.docx]

**Overview of Workshop One**

Anticipated length of time for workshop: ~2.5 hours (maybe late arrivals)

1. Introductions: 5 mins
2. Overview of the session: 5 mins
3. Ice breaker: 5 mins
4. ‘Post-it’ exercise – questions for a health care professional: 15-20 mins
5. Ice breaker: 5 mins
6. Review of HPV vaccine materials: 15-20 mins
7. Break and refreshments: 25 mins
8. Video(s) about the HPV vaccine: 20-25 mins
9. Ice breaker: 5 mins
10. Role play exercise – peer interview: 15-20 mins
11. Feedback from young people: 5-10 mins
12. Summary and close: 5 mins

*Equipment required for face-to-face workshops*

- Refreshments

- Welsh / Scottish / English HPV vaccine leaflets

- Bag of Starburst (for ice breaker)

- Digital recorders x 2

- Device (e.g. laptop) to play preloaded videos

- Soft balls x 2 (for introductions)

- Name labels

- Coloured pens / markers

- Post-it notes

- Paper

- Cards with questions young people have previously asked about the HPV vaccine

- Gift vouchers for participants (also for online participants)

*Online workshops*

If the workshop is delivered online, it would be preferable for two workshops with no more than three young people to allow all young people to engage with the process. Wherever possible, ask young people to sit near to the screen and talk loudly otherwise it may be difficult to transcribe.

1. **Introductions (5 mins)**

Depending on the set up, young people may or may not know each other. Introductions are helpful for researchers to get to know young people before the session starts and if young people do not know each other. Feedback from young people suggested they would feel more comfortable talking with researchers with their friends present. It seems likely that workshops organised in youth organisations will be with young people, who already know each other.

*N.B. Select either one of the following introduction tasks.*

- *A) Introductions: My name is? (online or face-to-face, can be used if young people already know each other)*

Go around the group and ask each workshop participant to state their name, who they are, and attach an adjective that not only describes them, but also starts with the same letter of their name (e.g. Talkative [], Happy []). Refer to all participants by these names for the rest of the workshop. If young people are attending a face-to-face workshop, provide young people labels with these names.

This has been helpful during the pilot session to gain some insight as to how the young people are feeling that day. Gives an opportunity for follow-up questions with young people.

- *B) Introductions: Time bomb (only if face-to-face, best if young people do not know each other)*

Invite participants to sit facing inwards in a circle. A small foam or tennis ball will be the ‘bomb’. A participant is selected to start with the ‘bomb’. They call out someone’s name and throw the ‘bomb’ to them. That person has two seconds only to say someone else’s name and throw the ‘bomb’ to them.

Participants leave the game if they hold the ball for too long, don’t say someone’s name before they throw, or the throw cannot be caught by the next person. Upgrade to two ‘bombs’.

1. **Overview of the session (5 mins)**

- *Explain the purpose of EDUCATE co-production study to young people*

We have found in our previous research that young people have unanswered questions about the HPV vaccine. We think it’s important that young people have an opportunity to find out about the HPV vaccine to help them decide whether they want the vaccine or not. In this project, we want to work with young people to find out what are the important things about the HPV vaccine young people want to know. We will work with film makers to create a series of films which we hope will improve the way young people find out about the HPV vaccine. We will work collaboratively with young people throughout this study, by working with young people to create the films and getting feedback from young people.

- *Explain the purpose of the workshop to young people*

As part of the study we will carry out a number of workshops with different groups of young people to get feedback on the films that we are developing. For this workshop, we would like to find out what are the most important things that we should cover before we start the filming. We will use the feedback you give us today to help us produce films that contain the information that is most relevant to young people.

Remind young people we are trying to find out what they think – we won’t be offended if they are critical or don’t like the materials that we share with them.

1. **Ice breaker (5 mins)**

*Select an ice breaker based on delivery mode of workshop and whether young people know each other or not.*

1. **Post it note exercise – Questions for a Healthcare professional (15-20 mins)**

Before the exercise starts, ask young people if they know about any of the vaccines that are given in secondary schools? Have they had any of these vaccines? Encourage young people who have been vaccinated to talk about their experiences in school.

- *Provide an outline of the HPV vaccine to young people*.

The HPV vaccine is offered to young people aged 12 to 13 in schools. It protects young people from being infected with a virus called the human papillomavirus, or HPV. Sometimes this virus can lead to the development of cancer. This could be cervical cancer that affects women. It also causes cancers affecting the throat, neck, and penis in men.

- *Ask the group to consider the following question:* ***‘If you were talking to an immunisation nurse or doctor about having the HPV vaccine, what questions would you ask?’***

*If face-to-face:*

- Give participants two minutes to brainstorm and write their responses on post-it notes.
- Encourage participants to write each response on a different post-it note.
- Ask young people to put all the post-it notes on a flip chart or wall.
- Ask one of the participants to organise the post-it notes into groups of similar questions.
- Do other young people agree with the groups?
- Are there any outliers?
- Encourage other participants to change the assignment of groups or add more ideas as necessary.

*If digital (may be flexibly delivered e.g. all separately online, at youth organisation via platform):*

- Give participants two minutes to brainstorm and to think/write/verbalise their responses. Ensure young people have paper/post-it’s to write their response if they wish.
- As a group, ask young people to communicate all of their suggestions (either verbally or by instant message depending on setup).
- The researcher could share the screen with young people. As young people provide feedback, write responses in text boxes within the word document.
- Participants should be able to see the document. Researcher should ask young people to suggest ways the text boxes should be grouped, or which questions are the most important.
- Do other young people agree with the groups?
- Are there any outliers that we need to consider?
- Encourage other participants to change the assignment of groups or add more ideas as necessary.

*For digital and face-to-face delivery*

Bring to the session cards with questions that young people have asked during pilot workshops and pilot schools sessions:

- Is the vaccine optional or compulsory?
- Will the vaccine once injected change you in anyway?
- Is there a chance that the vaccine can go wrong?
- Is there a cure for HPV cancers?
- Could HPV affect me at this age?
- What is inside the vaccine?
- What does the vaccine do to you?
- Are there any side effects from the vaccine?
- What are symptoms of HPV?
- If there aren’t any symptoms of HPV how do you find out you have it?
- If you get the cancer can you cure it?
- Does HPV happen when having sex?
- What will happen if the swelling doesn’t go away?
- When you get the vaccine what is the most serious side effect?
- Is there any signs of the cancer HPV?
- I heard that lots of people faint. Is that a side-effect?
- How will you know that you have cancer on your penis?
- Does treatment of cancer on your penis hurt?

Ask young people if they think these are important questions as well. If any of the questions are the same as questions young people in the workshop have come up with, group them together.

To finish off the session, ask young people to prioritise the most important questions to ask a healthcare professional. Explain to the young people at the end of the session we will revisit the questions they have if they haven’t been answered throughout the session. Thank young people for their input.

1. **Ice breaker (5 mins)**

*Select an ice breaker based on delivery mode of workshop and whether young people know each other or not.*

1. **Review of HPV vaccine materials (15-20 minutes)**

Provide young people with copies of the HPV vaccine leaflets for the English, Welsh and Scottish vaccination programmes.

Group 1 (HF): English and Welsh leaflets

Group 2 (TC): English and Scottish leaflets

*If face-to-face*

Break out in small groups of 2-3 young people with a researcher assigned to each group. Ensure there is a dictaphone recording near each group. Provide young people with paper and pens to write their responses. Ask young people to provide feedback about the leaflets. Ensure each leaflet is reviewed by at least one group. There may not be enough time or concentration for groups to provide feedback on all the leaflets.

*If digital*

Provide young people with paper and pens to write their responses. Ask young people to provide feedback about the leaflets. Ensure each leaflet is reviewed by at least one group. There may not be enough time for each young person to provide feedback on all the leaflets.

- What did they like about it?
- What did they not like about it?
- What is the most important thing they would change?
- What are the most important messages from the leaflet?
- Is there anything they would prefer to see more of?
- Are there any words they didn’t understand?
- Do they young people look like the young people they spend time with?
- Do they prefer images of young people in school uniforms?

1. **Break and refreshments (25 minutes)**

Can be introduced earlier if needed.

**8. Videos about the HPV vaccine (20-25 minutes)**

*N.B. Have video set up to play on laptop or other electronic device as well.*

*Video one*

Explain to the group that they will watch a video that was made for young people about having the HPV vaccine.

Group 1 (HF)

Explain to the group that they will watch a video which was made in Australia to help young people find out about having the HPV vaccine:

- <https://www.health.gov.au/resources/videos/hpv-animation-video-for-students>

Group 2 (TC)

Explain to the group that they will watch a video which was made in the UK to help young people find out about having the HPV vaccine:

- [https://www.healthforteens.co.uk/health/immunisation/video-5-rumours-about-the-hpv-vaccine/](https://www.healthforteens.co.uk/health/immunisation/video-5-rumours-about-the-hpv-vaccine)

Ask young people to provide feedback on the video.

- What did you like about it?
- What did you not like about it?
- Do you think this would help answer any of the questions that you had having the HPV vaccine?
- Did you like the graphics?
- What could be improved?
- Is there anything else you would like to know before you got the HPV vaccine?

*Video two*

Group 1 (HF)

Explain to the group that they will watch a video which was made in Canada to help motivate young people to have the HPV vaccine:

- <https://immunizebc.ca/hpv>

Group 2 (TC)

Explain to the group that they will watch a video made in New Zealand to explain why young men need the vaccination as well as young women.

- [https://www.health.govt.nz/your-health/healthy-living/immunisation/immunisation-older-children#videos](https://www.health.govt.nz/your-health/healthy-living/immunisation/immunisation-older-children#videos )

Ask young people to provide feedback on the video:

- What did you like about it?
- What did you not like about it?
- Do you think this looks like the vaccination sessions that have taken place at your school? (behaviour, ethnicity)
- What could be improved?
- Is there anything else you would like to know before you got the HPV vaccine?

Encourage young people to compare and contrast the parts of the videos that they preferred.

1. **Ice breaker (5 mins)**

*Select an ice breaker based on delivery mode of workshop and whether young people know each other or not.*

**10. Role play exercise – peer interview (15-20 mins)**

*Ask the group to consider the following question:* ***‘If you were talking to an older young person or your brother or sister about having the HPV vaccine, what questions would you ask?’***

- Ask participants to work in pairs to brainstorm and write their responses on post-it notes
- Encourage participants to write each response on a different post-it note.
- Create a role play where one young person asks the other young person the questions. This could work better in small outbreak groups, facilitated by researcher and youth workers.
- Ask for volunteers to act as the interviewer and interviewee.
- Ensure the dictaphone is near the young people at this stage
- Researchers should write the questions on a flip chart

At the end of the session, refer back to the questions/post-it notes developed from the previous first activity. Provide the young people with cards with questions asked by other young people in previous session (see below). Ask young people if there are any of these questions that they would prefer to ask another young person instead of the healthcare professional. Encourage young people to change the assignment of groups or add more ideas as necessary.

- I don’t want to take it, but I know I should. What should I do?
- Are you allowed to drink and eat after?
- When you are ill on the day in Year 8, will you have to do 2 doses in Year 9?
- Where do you get the jab on the body?
- What does the liquid feel like going in?
- Does it hurt?
- If your injection place becomes infected or itchy what should you do?
- Can you go to the doctors and get an injection instead of having one at school?
- Can I wear earphones when I’m having the vaccine?
- When are we getting the vaccine?
- Do I have to have the vaccine in school?
- What if I am sick on the day of the session?
- Do I wear a short sleeve for the whole day?
- Will I be vaccinated in front of everyone?
- What arm do I have the HPV vaccine in?
- When do we have the vaccine?

**11. Feedback from young people (5-10 mins)**

- Has any of this been enjoyable?
- Has any of this not been enjoyable? *(remind young people it’s okay to be honest!)*
- What has not worked well?
- Any suggestions of different icebreakers they have enjoyed in the past?

**12. Summary & close (5 mins)**

- Summarise the main points of the workshop
- Answer any questions on the post-it notes that young people still have
- Restate how the information generated in the session will feed into future filming
- Thank everyone for their participation
- Provide young people with Love to Shop gift vouchers/codes.
